# Comparative and phylogenetic analyses of the chloroplast genome reveal the taxonomy of the *Morus* genus

**DOI:** 10.3389/fpls.2022.1047592

**Published:** 2022-11-24

**Authors:** Qiwei Zeng, Miao Chen, Shouchang Wang, Xiaoxiang Xu, Tian Li, Zhonghuai Xiang, Ningjia He

**Affiliations:** State Key Laboratory of Silkworm Genome Biology, Southwest University, Chongqing, China

**Keywords:** Mulberry, Chloroplast genome, Phylogenetic tree, Taxonomy, Morus alba

## Abstract

Mulberry (genus *Morus*) is an economically important woody plant with an altered ploidy level. The variable number of *Morus* species recognized by different studies indicates that the genus is in need of revision. In this study, the chloroplast (CP) genomes of 123 *Morus* varieties were *de novo* assembled and systematically analyzed. The 123 varieties represented six *Morus* species, namely, *Morus alba*, *Morus nigra*, *Morus notabilis*, *Morus rubra*, *Morus celtidifolia*, and *Morus serrata*. The *Morus* CP genome was found to be 158,969~159,548 bp in size with 125 genes, including 81 protein coding, 36 tRNA, and 8 rRNA genes. The 87 out of 123 mulberry accessions were assigned to 14 diverse groups with identical CP genome, which indicated that they are maternally inherited and share 14 common ancestors. Then 50 diverse CP genomes occurred in 123 mulberry accessions for further study. The CP genomes of the *Morus* genus with a quadripartite structure have two inverted repeat (IR) regions (25,654~25,702 bp) dividing the circular genome into a large single-copy (LSC) region (87,873~88,243 bp) and small single-copy (SSC) region (19,740~19,994 bp). Analysis of the phylogenetic tree constructed using the complete CP genome sequences of *Morus* revealed a monophyletic genus and that *M. alba* consisted of two clades, *M. alba* var. *alba* and *M. alba* var. *multicaulis*. The Japanese cultivated germplasms were derived from *M. alba* var. *multicaulis*. We propose that the *Morus* genus be classified into six species, *M. nigra*, *M. notabilis*, *M. serrata*, *M. celtidifolia*, *M. rubra*, and *M. alba* with two subspecies, *M. alba* var. *alba* and *M. alba* var. *multicaulis*. Our findings provide a valuable resource for the classification, domestication, and breeding improvement of mulberry.

## Introduction

Mulberry (*Morus* L., Moraceae) (Group, 2009) comprises a variable number of species, with the first 7 described by Linnaeus (1753). The traditional taxonomy of *Morus* is often based on minor morphological differences (Gardner et al., 2021). Various researchers identified 5, 8, 13, 16, 24, and 35 *Morus* species using morphological and/or molecular methods (Bureau, 1873; Koidzumi, 1930; Hotta, 1954; Zhou and Gilbert, 2003; Zeng et al., 2015; Jain et al., 2022). The classification of the *Morus* genus based on morphology did not truly reflect the phylogenetic relationships (Jiao et al., 2020). In 2015, we proposed eight species in the *Morus* genus on the basis of ITS (internal transcribed spacer) sequences, which were recently verified by an analysis of population genetics (Jiao et al., 2020). A recent investigation of Moraceae revealed that the genus *Morus* is a monophyletic group without *M. mesozygia* and *M. insignis*, and the delimitation of *M. alba* may be worth further investigating (Gardner et al., 2021). However, the genome-based taxonomy of the genus *Morus* remains unexplored (Jiao et al., 2020).

Since the first mulberry genome (*M. notabilis*) was published (He et al., 2013), six other mulberry genomes, including chromosome-level genomes, have been reported (Jiao et al., 2020; Muhonja et al., 2020; Jain et al., 2022; Xia et al., 2022; Xuan et al., 2022), which represent excellent reference genomes for genomic and population analyses of mulberry resources. Different chromosome numbers (14, 28, 35, 42, 49, 56, 84, 112, 126, and 308) with various ploidy levels have been reported in mulberry (Tikader and Kamble, 2008; Xuan et al., 2022). For example, black mulberry (*M. nigra*) is a polyploid with 308 chromosomes (Agaev and Fedorova, 1970; SB et al., 1990), and *Morus serrata* is a natural polyploid with 56 or 84 chromosomes (SB and Rajan, 1989). Variable ploidy levels are often observed in white mulberry (*M. alba*) (Xuan et al., 2019). The variable ploidy levels in *Morus* adversely affect population genetic analyses for taxonomic purposes. Mulberry has been cultivated by farmers for over 5000 years (He et al., 2013), and many varieties or cultivars have been generated by natural and artificial breeding selection. More than 2600, 1500, and 1120 mulberry germplasm resources were recorded in China, Japan, and India, respectively (Vijayan et al., 2011; Vijayan and B.da Silva, 2011). The evolutionary relationships of these cultivars or varieties remain unclear.

Chloroplast, a plant cell organelle with its own genome, is essential for the growth and development of plants. Compared with the large nuclear genome, chloroplast genomes are smaller. CP genomes with their numerous advantages for plant phylogeny reconstruction, including a relatively conserved rate of evolution and usually uniparental inheritance, provide an important resource for elucidating morphological evolution (Gitzendanner et al., 2018; Li et al., 2021; Hua et al., 2022). CP genomes also provide critical insights into historically difficult relationships of the major angiosperm subclades (Moore et al., 2007; Moore et al., 2010; Stull et al., 2015; Sun et al., 2016; Li H-T. et al., 2019). We proposed that the CP genomes possibly provide insights into the evolution and taxonomy of the *Morus* genus. Since the CP genome of *M*. *indica* var. K2 was first obtained (Ravi et al., 2006), those of *M*. *mongolica* (Kong and Yang, 2016), *M*. *alba* var. *atropurpurea* (Li et al., 2016), *M*. *notabilis* (Chen et al., 2016), *M*. *alba* var. *multicaulis* and *M*. *cathayana* (Kong and Yang, 2017), *M. alba* (Luo et al., 2019; He et al., 2020), etc., have been reported. However, a large-scale comparative analysis of the CP genome across the *Morus* genus has not yet been conducted.

Therefore, the purpose of this study is to conduct a large-scale comparative genomic analysis of *Morus* CP genomes and reconstruct phylogenetic tree based on CP genomes to explore the taxonomy of genus *Morus*. The evolutionary relationships of mulberry accessions were also explored based on their CP genomes. These results provide an important information for the classification, domestication, and breeding improvement of mulberry.

## Material and methods

### Sample collection and sequencing

*Morus serrata* was collected from Jilong, Tibet Autonomous Region, China, and propagated at the Mulberry Germplasm Nursery at Southwest University. *Morus celtidifolia* was identified by Professor Elizabeth Makings from Arizona State University, USA. *Morus notabilis* was collected from a pristine forest in Ya’an, Sichuan Province, China. *Morus yunnanensis* was obtained from the Institute of Sericulture and Apiculture, Yunnan Academy of Agricultural Sciences, Mengzi, Yunnan Province, China. *Morus nigra* was collected from Yutian County, Xinjiang Uygur Autonomous Region, China. Other samples were obtained from the Mulberry Germplasm Nursery at Southwest University, China. For each sample, 10 µg genomic DNA was extracted from young leaves according to a standard cetyltrimethylammonium bromide protocol for the subsequent construction of sequencing libraries. Specifically, sequencing libraries with an average insert size of 350 bp were constructed according to the Illumina standard protocol, after which they were sequenced by BGI-Shenzhen (Shenzhen, China) using the Illumina HiSeq XTen or MGISEQ-2000 platform (Illumina, San Diego, CA, United States) to generate 150-bp paired-end reads. The raw data of 35 samples have been deposited in the CNGB Sequence Archive of the China National GeneBank Database (CNGBdb) under accession number CNP0001407. Using these data and publicly available genomic data downloaded from the NCBI or CNGB database, the *Morus* CP genomes were studied (**Supplementary 1**). The adapters and low-quality sequences were removed using the program fastp (Chen et al., 2018) from the raw reads to obtain clean reads for the subsequent analyses.

### *De novo* assembly and annotation of the chloroplast genome

NOVOPLasty (version 4.3) (Dierckxsens et al., 2017) and GetOrganelle (v1.7.6.1) (Jin et al., 2020), which were developed for the *de novo* assembly of organelle genomes, were used for assembling CP genomes. For NOVOPLasty, default parameters were applied, with the following exceptions: read length (100 or 150), genome range (150,000–170,000), and K-mer optimized. For GetOrganelle, default parameters were applied, with the following exception: the heyebai chloroplast genome sequence (KU981119) as a reference sequence. Because the two haplotypes are present in the same proportion in a cell (Wang and Lanfear, 2019), we then selected the haplotype with the same SSC orientation as that in the CP genome sequences for further analyses.

The complete CP genomes were annotated in CPGAVAS2 (Shi et al., 2019) with default parameters. The ambiguous gene positions were manually corrected by NCBI BLASTN searches. All transfer RNA genes were confirmed on the tRNAscan-SE 2.0 web server with default settings (Lowe and Chan, 2016). Their high-quality graphical maps were drawn by OGDRAW (Lohse et al., 2013) with default parameters. All annotated chloroplast genome sequences were submitted to GenBank through BankIt (https://www.ncbi.nlm.nih.gov/WebSub/index.cgi).

### Comparative chloroplast genome analysis

The mVISTA program (http://genome.lbl.gov/vista/mvista/about.shtml) was employed to determine the differences in the whole chloroplast genomes of *M. notabilis* (MK211167), *M. serrata* (MT154044), *M. celtidifolia* (MT154045), *M. alba* var. *multicaulis* (OP153908), *M. alba* var. *alba* (OP153917), *M. nigra* (OP153918), *M. alba* var. *indica* (OP153922), and *M. rubra* (OP161259), in the Shuffle-LAGAN mode with *M. notabilis* as the reference genome.

### Sequence divergence analysis

MAFFT v7.455 software (Katoh and Standley, 2013) was employed to align the CP genomes of 50 *Morus* accessions. DnaSP v5.10 software (Librado and Rozas, 2009) was used to identify rapidly evolving molecular markers with a sliding window analysis (window length and step size set as 500 and 250 bp, respectively). The R package ggmsa (Zhou et al., 2022) was used to visualize multiple sequence alignments of target regions from 50 CP genomes.

### Phylogenetic analysis

We assigned mulberry accessions with the identical CP genomes to a group, therefore 87 out of 123 mulberry accessions were assigned to 14 different groups and other 36 accessions were classified to an unclassified group. In 14 different groups, each group randomly selected a representative CP genome, together with other 36 CP genomes from the unclassified group, to form 50 CP genomes for phylogenetic analysis. The CP genome sequences of *Broussonetia papyrifera* (*Broussonetia* genus, GenBank: MZ662865), *Ficus carica* (*Ficus* genus, GenBank: KY635880) and *Morus mesozygia* (*Afromorus* genus, GenBank: MZ274134) (Gardner et al., 2021) were selected as outgroups. All complete CP genome sequences were aligned using MAFFT v7.455 (Katoh and Standley, 2013), and the alignments were trimmed with trimAl v1.4.rev15 (Capella-Gutiérrez et al., 2009). IQ-TREE 2.0 (Minh et al., 2020) was employed to construct a maximum likelihood (ML) phylogenetic tree with 1000 bootstrap replicates. Finally, the phylogenetic tree was edited using iTOL 5.0 (https://itol.embl.de/) (Letunic and Bork, 2019) and Adobe Photoshop^®^ CC (Adobe Systems Inc., California, U.S.A.).

## Results

### Features of the *Morus* species chloroplast (CP) genomes

**Figure 1:**
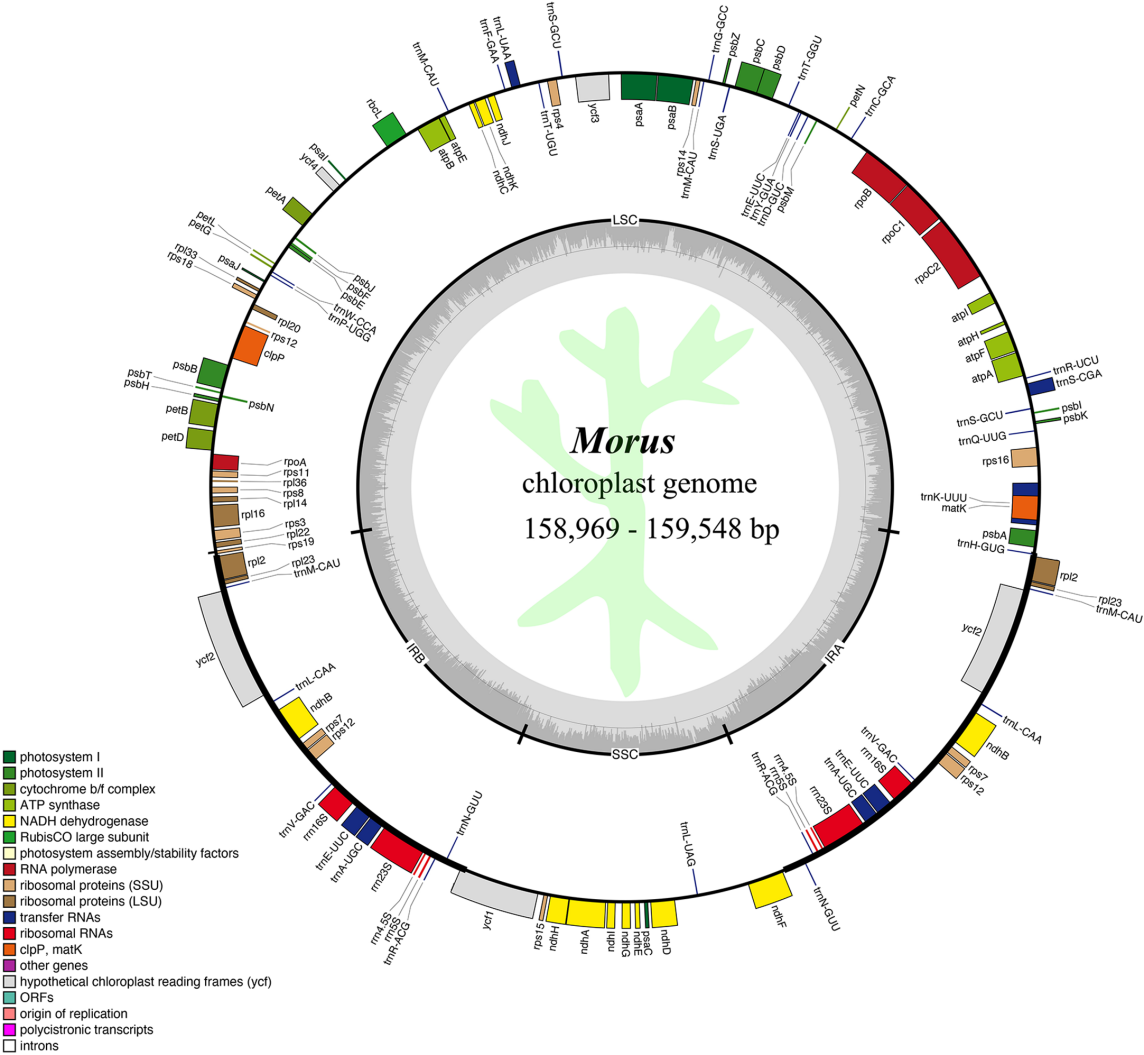
Genome map of the average *Morus* CP genome studied in this work. The inner circle represents the quadripartite structure, with an LSC, an SSC, and inverted repeat regions (IRA and IRB) and GC content shown in dark gray and AT content shown in light gray. The external circle represents gene content, with those outside the circle transcribed counterclockwise, while those located inside are transcribed clockwise. Genes are colored according to functional groups defined in the legend shown in the bottom left. The script in light green represents Jiaguwen mulberry.

We *de novo* assembled 123 complete CP genomes of *Morus* species with sizes ranging from 158,969 to 159,548 bp (**Figure 1**; **Supplementary 1**). These CP genomes display a typical circular quadripartite architecture, with an LSC region (87,873~88,243 bp) and an SSC region (19,740~19,994 bp) separated by two inverted repeat (IR) regions (25,654~25,702 bp) (**Supplementary 1**). All CP genomes showed similar total GC contents (ranging from 36.13% to 36.21%). The largest length change in CP genome sequences occurred upstream of psbA of *M. alba* var. *indica* with a 135 bp missing sequence, which led *M. alba* var. *indica* to have the shortest CP genome (**Supplementary 1**).

The mulberry accessions with same CP genomes were assigned to a group, 87 out of 123 accessions were assigned to 14 diverse groups and other 36 accessions with different CP genomes were assigned to an unclassified group (**Table 1** and **Supplementary 1**), which indicated that these 87 mulberry accessions were inherited maternally and shared 14 common ancestors. Of them, the largest group, ONE, comprises 28 *Morus* accessions, including 9 Japanese mulberry accessions and 9 Husang (*M. alba var. multicaulis*) accessions (**Table 1**). The nine Husang germplasms include heyebai (Husang-32), Jiantouheyebai, Husang-103, Husang-26, Husang-37, Husang-10, and Husang-60. Group TWO consists of 13 *Morus* accessions, including 12 Indian mulberry samples. Group THREE consists of 10 different Chuansang (*M. notabilis*) samples collected in a pristine forest around Ya’an, Sichuan Province. Group SIX comprises 4 samples, including one maternal parent and its three hybrid progenies. Group EIGHT comprises 4 Japanese samples, Yamasou-159, Ichinose, Fengwei-Ichinose, and Shinichinose. In 14 different groups, each group randomly selected a representative CP genome, together with other 36 CP genomes from the unclassified group **Table 1**, to form 50 diverse CP genomes occurred in 123 mulberry accessions for further analysis. And these 50 CP genomes were deposited in NCBI with GenBank accession numbers OP153890-OP153924, OP161257-OP161267, OP380682-OP380686, MK211167, MT154044, MT154045, and OP142713 for further analysis (**Supplementary 1**).

**Table 1:** Group information of the 123 mulberry germplasm accessions.

Groups	Sample names of mulberry germplasm accessions
ONE	Heyebai, Jiantouheyebai, Husang-103, Heyebai-reseq, Husang-26, Husang-37, **Nezumigaeshi**-2, **Kenmochi**, Tuosang-25, **Kumonryu**, **Kenmochi-2**, **Gunmaakagi**, **Shuangtou-Kenmochi**, Tieyezi, Liyeda, Husang-10, Husang-60, zhangyasang, Heyebai, **Kairyouwasejumonji**, 6071, Chaoxiansang, Guizhoumaosang-31, **Kenmochi**, Gelujiya, Huosang-1, NXS, **Dateakagi**
TWO	kanva2, SL2, Mulberry-MJ049, Mulberry-MJ036, Mulberry-MJ038, Mulberry-MJ033, Mulberry-MJ041, Mulberry-MJ039, Mulberry-MJ047, Mulberry-MJ035, kanva2, Mulberry-MJ042, Mulberry-MJ032
THREE	*M. notabilis*_2, *M. notabilis*_4, *M. notabilis*_5, *M. notabilis*_reseq, wild_mulberry_S16, f_CHS_1, m_CHS_1, m_CHS_3, f_CHS_2, m_CHS_2
FOUR	Shidian-6, Dazhongsang, Linxianlusang, RL0424, Shaqi-1, Huanglutou
FIVE	Huasang, Cangxisang, Taiwanchangguosang, SL1, Mulberry-MJ044, Mulberry-MJ045
SIX	Hybrid-13, Hybrid-n13, 12, Lunjiao109x12
SEVEN	Chuizhisang, Chuizhisang, Chuizhisang, ZCS
EIGHT	**Yamasou-159**, **Ichinose**, **Fengwei-Ichinose**, **Shinichinose**
NINE	*Morus multicaulis*, **Shaansang305**
TEN	Huanggelu, Heigelu
ELEVEN	*Morus yunnanensis*, *Morus yunnanensis*
TWELVE	Lunjiao109_G, Lunjiao109_C
THIRTEEN	Xiongyue-107, Jizhuasang
FOURTEEN	T1, Tseed
UNCLASSIFIED	Pisang-2, Yun-7, *Morus macroura*, *Morus australis*, *Morus wittiorum*, Yun-6, **Kairyonezumigaeshi**, Liquan-1, Qinbasang, JiLongSang, *Morus nigra*, *Morus celtidifolia*, Zhaisang-1, Jiaqing-9, Guapiaosang, Dabaie, Shuangcheng, Heilujiesang, Jainzhizi, **Yamasou -106**, Basailuona, Chenkou-3, JPZ, Tianquan-12, *Morus rubra*, *Morus cathayana*, Gui-23, Kangqin283, Hongye, Baiyuwang, Zhenzhubai, Shuisang, WG120_1, Husang-192, Pisang, Luohang-5

Sample names in bold are from Japanese mulberry germplasm accessions. Lunjiao109_G and Lunjiao109_C represented samples collected in Guangdong province and Chongqing province, China, respectively.

We annotated 125 genes in each *Morus* CP genome, consisting of 81 protein-coding genes, 8 rRNAs, and 36 tRNAs (**Figure 1**). In detail, 17 duplicated genes in the IR region were identified, including 6 protein-coding genes, 4 rRNA genes, and 7 tRNA genes. Ten protein-coding genes and 1 tRNA gene exist in the SSC region, while 59 protein-coding genes and 21 tRNA genes are present in the LSC region. All annotated genes were concatenated into a supermatrix, whose sizes in the 50 CP genomes ranged from 109,073 to 109,159 bp (**Supplementary 1**). Twenty-six out of 50 supermatrices showed identical sequences in ten separate groups (**Supplementary 2**). Of them, the largest three groups contained five, four, and three members, respectively, indicating high conservation.

There were 22 genes containing introns in all *Morus* CP genomes (**Supplementary 3**). Among these genes, 7 are tRNA genes, and 15 are protein-coding genes. Most genes have only a single intron, whereas the clpP and ycf3 genes contain two introns (**Supplementary 3**). rps12 is a trans-splicing gene composed of three exons, containing one 5’ exon located in the LSC region and two 3’ exons located in the IR region (**Figure 1**). Compared to other intron-containing genes, the trnK-UUU gene embodied the matK genes and has the largest intron (2,553-2,563 bp) (**Supplementary 3**).

### Similarity analysis and nucleotide diversity of CP genomes

**Figure 2:**
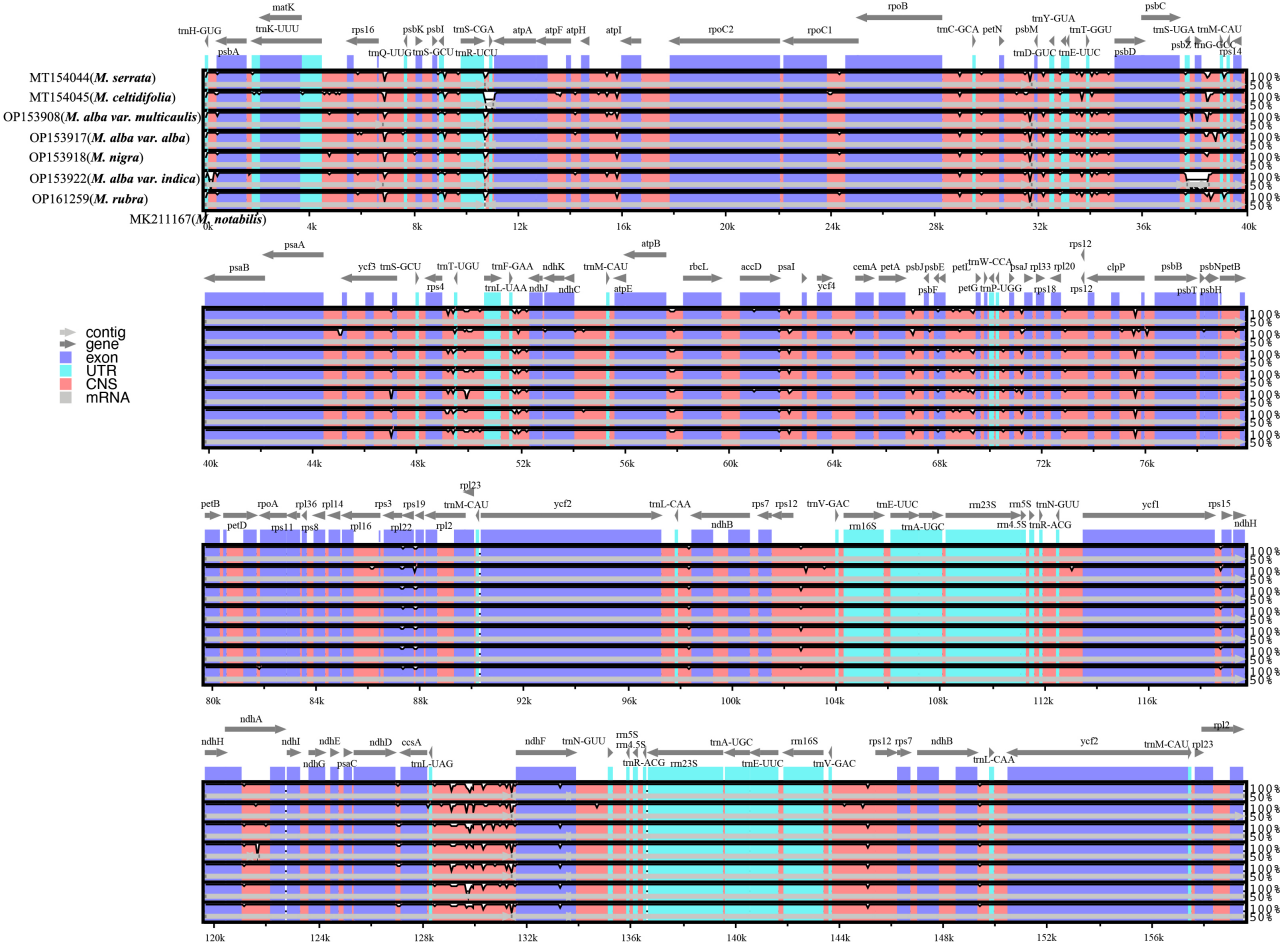
Sequence identity plot comparing the chloroplast genomes of six *Morus* species with *M. notabilis* as a reference. The vertical scale indicates the percentage of identity, ranging from 50 to 100%. The horizontal axis indicates the coordinates within the CP genome.

**Figure 3:**
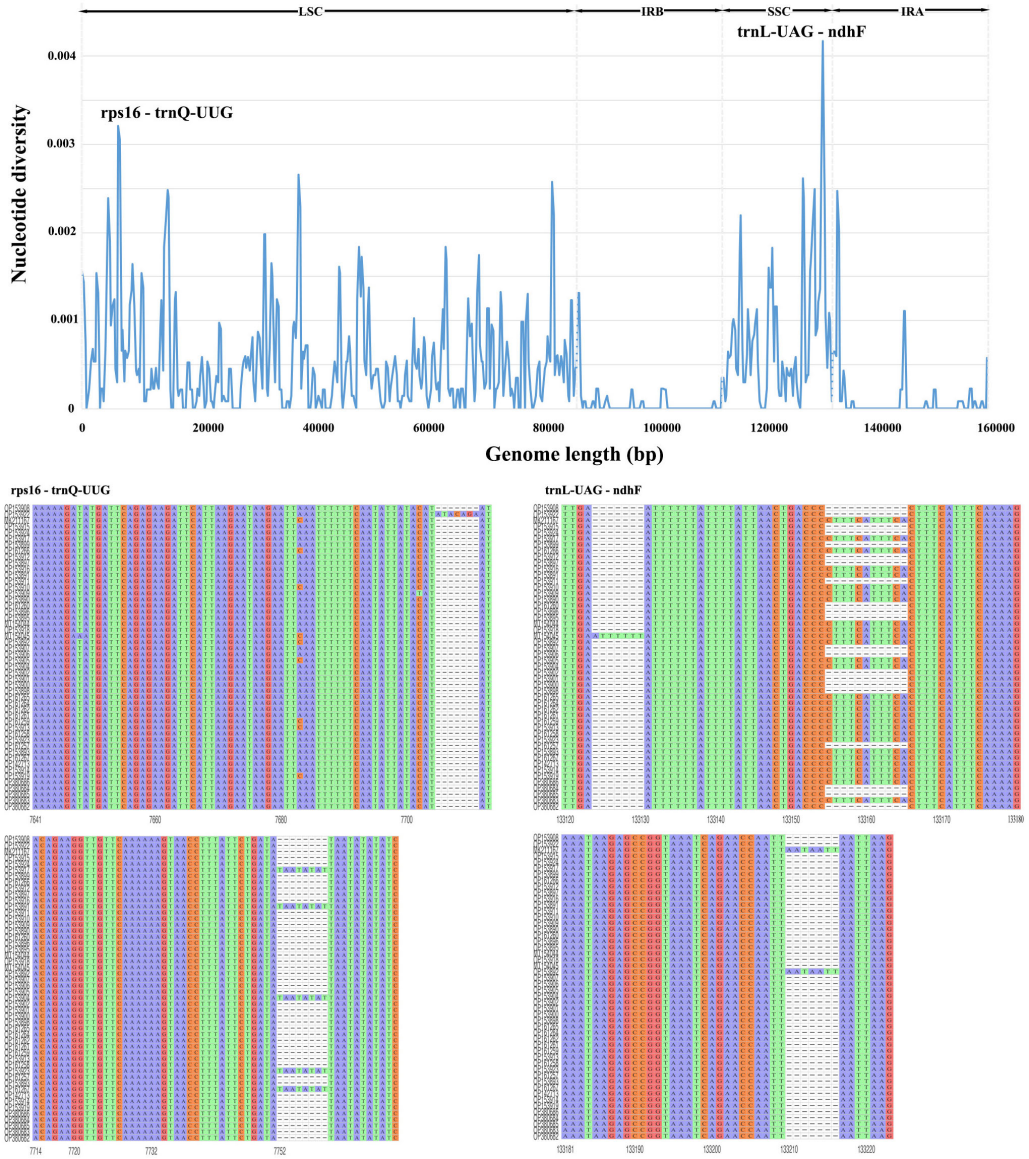
Comparative analysis of nucleotide variability by Pi values of the 50 CP genomes presented in a sliding window (window length: 500 bp; step size: 250 bp). X-axis: position of the midpoint of a window; Y-axis: nucleotide diversity in each window. The R package ggmsa was used to visualize the sequence alignment of two hotspot regions in the 50 CP genomes.

The sequence homology of the *Morus* species was investigated with *M. notabilis* as a reference using mVISTA software (**Figure 2**). The nucleotide variability (Pi) was calculated to further confirm the sequence variations (**Figure 3**). The *Morus* CP genomes were highly conserved and displayed similar structures and gene orders (**Figure 2**). The divergence level of the noncoding regions was higher than that of the coding regions. The protein-coding regions were highly conserved, and the ndhF genes displayed obvious polymorphism (**Figure 2**). The Pi values were rather low, ranging from 0 to 0.00442 among the 50 CP genomes, and 2 hotspot regions were identified with Pi >0.003 (rps16-trnQ-UUG and trnL-UAG-ndhF) (**Figure 3**). No highly variable loci were detected in the IR regions, and the nucleotide diversity values were significantly lower than those in the single-copy regions (**Figure 3**). Because of the highly conserved sequences, structure, and size of the CP genomes of *Morus*, no obvious hypervariable regions were noted (**Figures 2**, **3**). As a result, the complete CP genomes were considered to distinguish *Morus* species.

### Phylogenetic analysis

**Figure 4:**
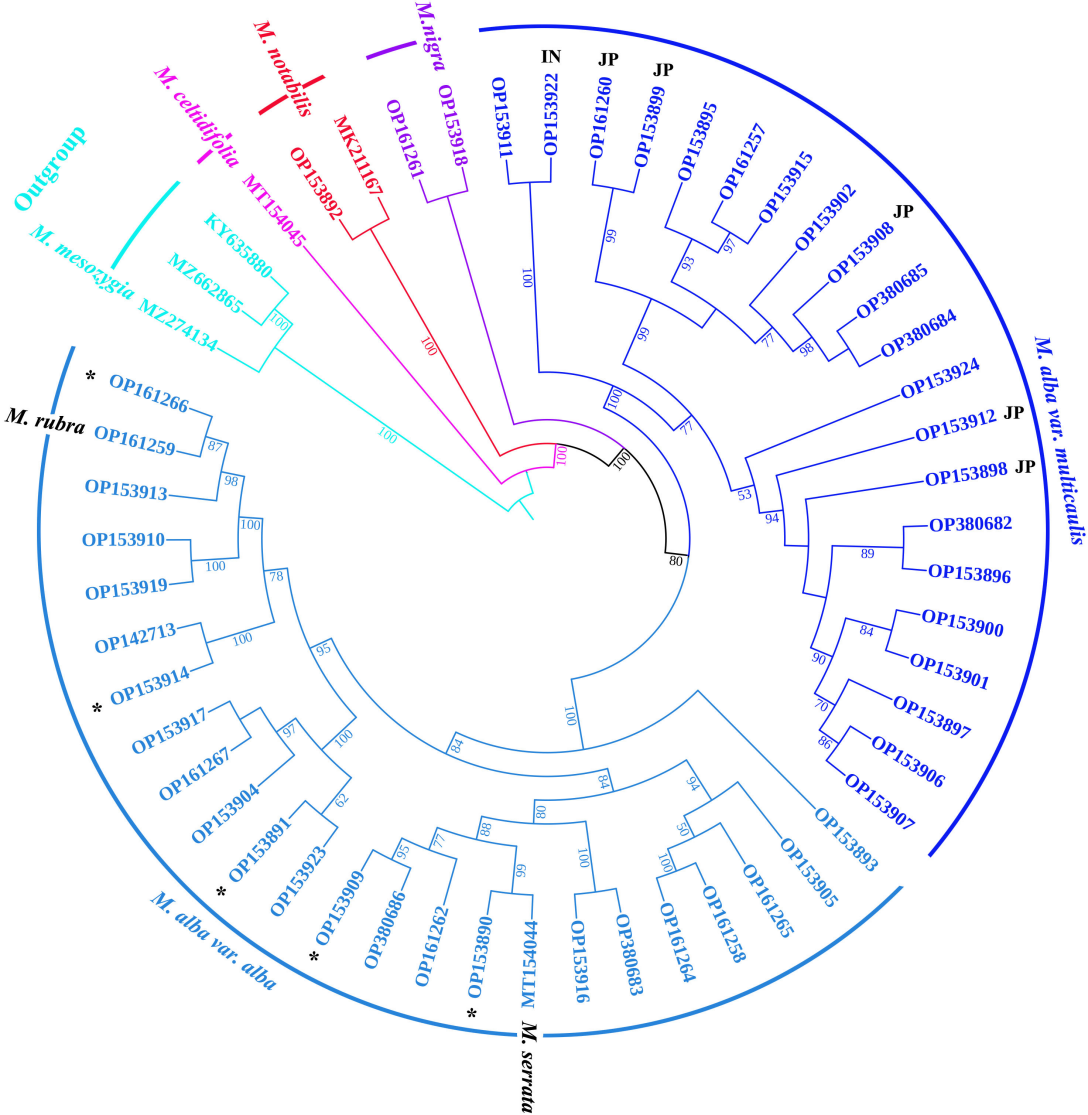
Phylogenetic relationships among *Morus* species based on their CP genomes with 50 *Morus* accessions and three outgroup genera (*Ficus*, *Broussonetia*, and *Afromorus*). The maximum-likelihood tree constructed with IQ-TREE2 is presented with complete CP genomes. The percentage of statistical support for the nodes is based on 1,000 bootstrap replicates. The black asterisks represent *Morus* accessions with mulberry fruit lengths over 4 cm. JP (Japan) and IN (India) in bold black represent mulberry accessions from Japan and India, respectively.

In this study, the 50 CP genomes representing 123 mulberry samples were utilized to explore the phylogenetic positions of *Morus* species. Because of the highly conserved coding-region sequences in the *Morus* CP genomes, the complete genomes were used to construct the maximum-likelihood (ML) tree. As illustrated in **Figure 4**, the phylogenetic tree was divided into five clades. Among them, Outgroup is a clade containing three different genera (*Ficus*, *Broussonetia*, and *Afromorus*) at the root. *Ficus carica* was clustered with *Broussonetia papyrifera*, which was a sister genus of *Morus*, indicating a close relationship between *Ficus* and *Broussonetia*. *Morus mesozygia*, an outgroup member, was recognized as a *Morus* species native to Africa and belongs to the *Afromorus* genus. *M. celtidifolia*, a species native to America, was an independent clade. *M. notabilis*, native to Sichuan Province, and *M. yunnanensis*, native to Yunnan Province, formed a clade. Black mulberry (*M. nigra*) was a clade. White mulberry (*M*. *alba*) was the most complex and largest clade and was further divided into two subclades, *M. alba* var. *alba* and *M. alba* var. multicaulis, indicating that there were two subspecies of *M. alba* species. The *M. alba* var. *multicaulis* subclade comprised three subgroups containing all Husang, *M. alba* var. *indica*, and Japanese mulberry accessions. The *M. alba* var. *alba* subclade contained three subgroups, including the red mulberry (*M. rubra*), *M. serrata*, and a wild mulberry collected in Tibet, China. In addition, the mulberry resources (*M. alba* var. Taiwanchangguosang, *M. alba* var. *shuisang*, *M*. *wittiorum*, *M. alba* var. Yun7 and *M. alba* var. Yun6) with long fruits (over 4 cm) were clustered in the *M. alba* var. *alba* subclade. At the same time, two *M. alba* var. *atropurpurea* germplasms (*M. alba* var. Lunjiao109 and *M. alba* var. Kanqin283) were placed in the *M. alba* var. *alba* subclade.

## Discussion

### *Morus* CP genome characterization

Maternally inherited CP genomes provide useful information for phylogenetic reconstruction (Bruun-Lund et al., 2017; Gitzendanner et al., 2018; Li et al., 2021; Wang et al., 2021; Zhang et al., 2022). Although some *Morus* CP genomes (Chen et al., 2016; Kong and Yang, 2016; Li et al., 2016; Kong and Yang, 2017; Luo et al., 2019; He et al., 2020) have previously been reported since the first was reported in 2006 (Ravi et al., 2006), there is a lack of large-scale comparative analysis of these genomes. Some *Morus* CP genomes deposited in the NCBI database were reference-based assemblies (Chen et al., 2016; Kong and Yang, 2016; Li et al., 2016), which may lack some useful information. For example, the CP genome of *M. notabilis* showed length differences between *de novo* assembly (GenBank: MK211167, size: 159,548 bp) and reference-based assembly (GenBank: KP939360, size: 158,680 bp). In addition, two indels over 40 bp were detected in the CP genome of the *de novo* assembly (GenBank: OP153912, size: 159,200 bp) compared with the reference-based assembly (GenBank: KU981119, size: 159,103 bp) using the same raw data. The performance of the reference-based assembly was dependent on the references employed (Scheunert et al., 2020). As a result, all *Morus* CP genomes were *de novo* assembled in this study, and *Morus* CP genomes of the reference-based assembly were not included. The size of *Morus* CP genomes ranged from 158,969 to 159,548 bp, which was larger than the first *Morus* CP genome (158,484 bp) (Ravi et al., 2006) and suggested that CP genome length in *Morus* was highly conserved. GC content is often considered an important indicator of species affinity (Chen et al., 2021), and the GC content of *Morus* CP genomes showed slight differences, ranging from 36.13% to 36.21%, which indicated high conservation in the *Morus* CP genomes. Twenty-two intron-containing genes out of 125 genes were detected in these CP genomes. Among them, the trnK-UUU gene embodied the matK genes and had the largest intron (over 2,500 bp), which has been reported in previous studies (Li X. et al., 2019; Souza et al., 2020; Ren et al., 2022). matK is a well-known gene that is often used for molecular identification and analysis of genetic relationships in plants (Hilu and Liang, 1997; Ramesh et al., 2022), including *Morus* (Venkateswarlu et al., 2012). As a result, over one hundred matK genes of the *Morus* genus have been deposited in the GenBank of the NCBI. Intron-containing genes often have important physiological functions; for example, the clpP gene is relevant to proteolysis (Shikanai et al., 2001), and the ndhB gene has an important role in mediating photosystem I cyclic electron transport (Shen et al., 2022). Therefore, introns in *Morus* CP genomes may be useful in terms of physiological function.

The largest group with identical CP genome contained 28 mulberry germplasm accessions, including 9 Husang accessions and 9 Japanese mulberry accessions. Husang (or Hu mulberry, *M. alba* var. *multicaulis*, with multicaulis meaning many stalks or branches), a well-known cultivar of domesticated mulberry, is widely distributed worldwide (Kenrick, 1839; Jiao et al., 2020). Heyebai, named Husang-32, is a control cultivar in the National Mulberry New Cultivar Identification Test (Jiao et al., 2020). The selection of Husang germplasms was mainly performed for open-pollinated seedlings after the Song Dynasty, and the excellent traits were retained by the asexual method (Jiao et al., 2020). Tens of Husang germplasms were obtained and recorded after selection over hundreds of years. Most of the cultivated mulberry varieties in Japan are derived from the three original species, namely, Yamaguwa (*Morus bombycis*), Karayumaguwa (*M. alba*), and Roguwa (*Morus lhou*) (Minamizawa, 1997; Muhonja et al., 2020), of which *M*. *lhou* and *M. bombycis* (Koidzumi, 1930) belong to *M. alba* (Zeng et al., 2015). Additionally, Karayumaguwa and Roguwa were introduced to Japan from Chinese *M. alba* species around A.D. 677 and A.D. 1873, respectively (Hotta, 1958). Therefore, those three original species in Japan belong to the *M. alba* species. Ichibei, Kenmochi, and Gunmaakagi are related to Yamaguwa, whereas Ichinose, Kairyonezumigaeshi, Nezumigaeshi, and Kairyowasejumonji are related to Karayumaguwa (Minamizawa, 1997). In this study, 9 Japanese samples shared the same progenitor with Husang, indicating that the 9 Japanese samples belonged to *M. alba* var. *multicaulis*. Kairyonezumigaeshi was selected from among Nezumigaeshi plants in 1907 (Muhonja et al., 2020), which is consistent with their identical CP genomes (**Table 1**), whereas Ichinose (group EIGHT) was isolated from Nezumigaeshi (group ONE) seedlings in 1901 (Yamanouchi et al., 2010; Smethurst, 2014; Muhonja et al., 2020) and showed a different CP genome (**Supplementary 1**), which implied that hybridization may increase the genetic diversity of CP genomes (Van Droogenbroeck et al., 2006; Daniell et al., 2016). In addition, in group EIGHT, 4 Japanese samples, Yamasou-159, Ichinose, Fengwei-Ichinose and Shinichinose, had common ancestors. Shinichinose, a hybrid variety derived from Ichinose (Muhonja et al., 2020), showed an identical CP genome with Ichinose, which is a typical character of maternal inheritance. In addition, group SIX showed another case of maternal inheritance because the maternal parent exhibited the same CP genome as its three hybrid progenies (**Table 1** and **Supplementary 1**). The autotriploid cultivar Shaansang305 (group NINE, 159,200 bp) induced from the diploid cultivar Shinichinose using colchicine (Liu et al., 2021) showed obvious differences from the CP genome of Shinichinose (group EIGHT, 159,219 bp) (**Supplementary 1**), which suggested that polyploidizations affected the DNA of the nucleus and chloroplast (Choopeng et al., 2019; Zhai et al., 2021).

Group TWO consisted of 13 different accessions, including 12 *M. alba* var. *indica*, which indicated that they had common ancestors. Group THREE consisted of 9 different *M. notabilis* trees located in regions with an approximately 10 km radius of the pristine forest in Ya’an, Sichuan Province, Southwest China, and one seedling germinated from an *M. notabilis* seed. Group ELEVEN contained two different *M. yunnanensis* trees collected on Dawei Mountain, Yunnan Province, Southwest China.

The genome size (158,969-159,548 bp) and GC content (36.13%-36.21%) of the *Morus* CP genomes exhibited slight differences, which indicated high conservation of *Morus* CP genomes. DnaSP (Librado and Rozas, 2009) and mVISTA software were employed to investigate the divergence of CP genomes of the *Morus* genus. The results showed high conservation of gene order and rather low Pi values (0-0.00442), and noncoding regions were more variable than coding regions. At the same time, over half of the supermatrices showed identical sequences (**Supplementary 2**), which further supported that the coding regions were highly conserved. Therefore, the complete CP genomes of *Morus* were considered to construct the phylogenetic tree for identifying *Morus* species.

### Phylogenetic analysis and taxonomical review of *Morus*

The 50 complete CP genomes of *Morus* were used to construct the ML tree for exploring the phylogenetic positions of the *Morus* species. It is clear that the genus *Morus* is monophyletic and is divided into five clades (**Figure 4**). Recently, *M. mesozygia* and *M. insignis*, native to Africa, which used to belong to the *Morus* genus, were eliminated from the *Morus* genus on the basis of phylogenetic analyses of supercontig sequences from 246 Moraceae samples (Gardner et al., 2021). Here, we also found that *M. mesozygia* did not belong to *Morus* because *M. mesozygia* was clustered with *Ficus* and *Broussonetia* (**Figure 4**).

*Morus yunnanensis*, similar to *M. notabilis* found in Southwest China with the same chromosome number, is a wild mulberry native to Yunnan Province, China (Xia et al., 2022). *Morus yunnanensis* was clustered with *M. notabilis* into a clade, indicating a close phylogenetic relationship (**Figure 4**). This close phylogenetic relationship between *M. yunnanensis* and *M. notabilis* was strongly supported by a phylogenomic tree (Xia et al., 2022). Combined with evidence from the nuclear genome and CP genome, we classify *M. yunnanensis* as belonging to *M. notabilis*.

Black mulberry (*M. nigra*), native to western Asia, is a Morus species with 308 chromosomes, which hinders the exploration of phylogenetic relationships based on the nuclear genome. It has been reported that *M. nigra* originated from *M. alba* (Agaev and Fedorova, 1970; Browicz, 2000; Lim., 2012), but molecular evidence is lacking. Fortunately, the CP genome is independent of the nuclear genome and is commonly used in phylogenetic studies. In our phylogenetic tree inferred from complete CP genomes, *M. nigra* displayed a close phylogenetic relationship with *M. alba* (**Figure 4**), which indicates that *M. nigra* originated from *M. alba*.

The largest clade in the phylogenetic tree of the *Morus* genus is the *M. alba* clade, comprising two subclades, *M. alba* var. *alba* and *M. alba* var. *multicaulis*, which is consistent with the taxonomy presented in the Flora of China (Zhou and Gilbert, 2003) and the phylogenetic tree based on domesticated mulberry accessions (Jiao et al., 2020). The *M. alba* var. *multicaulis* clade was divided into three subclades, including all Husang (*M. alba* var. *multicaulis*) and 16 Japanese mulberry samples, *M. alba* var. *indica* and other samples. Nine Japanese samples sharing the same CP genomes with Husang were clustered with seven other Japanese samples into the *M. alba* var. *multicaulis* clade, showing a close phylogenetic relationship, which indicated that these Japanese samples may have been derived from *M. alba* var. *multicaulis* through maternal inheritance. Recently, gene flow between Husang and Japanese samples was observed in the population structure analysis of mulberry accessions (Xia et al., 2022). Sixteen Japanese samples were clustered into two subclades, which was consistent with the findings of a previous report (Muhonja et al., 2020). Here, we provided molecular evidence that Japanese cultivated mulberry was derived from Chinese *M. alba* (Hotta, 1958; Jiao et al., 2020); therefore, we conclude that Japanese cultivated mulberry belongs to *M. alba* var. *multicaulis*. The subclade of *M. alba* var. *alba* contained red mulberry (*M. rubra*), *M. serrata*, *M. alba* var. *atropurpurea*, long-fruited mulberry germplasms, and other samples. Among them, *M*. *rubra*, a *Morus* species native to America, was clustered into *M. alba* var. *alba*, which may be triggered by common hybridization with *M. alba* (Burgess et al., 2008). It has been reported that *M. rubra* commonly hybridizes with *M. alba* and that *M. alba* potentially poses a threat to the existence of *M. rubra*, which leads to the endangerment of native *M. rubra* in America (Nepal and Wichern, 2013). In field observations, the direction of introgression of hybrids between *M. rubra* and *M. alba* was biased toward *M. alba* as the maternal parent (Nepal and Wichern, 2013). Mulberry germplasms with long fruits include *M. wittiorum* and *M. macroura*, which have been recognized as *M. alba* (Zeng et al., 2015). Here, we supplied new molecular evidence at the genome level. *Morus serrata* was classified as a species based on morphological taxonomy and molecular marker genes (Hotta, 1958; Zhou and Gilbert, 2003; Nepal and Ferguson, 2012; Zeng et al., 2015). However, in this study, *M. serrata* was clustered with *M. alba* var. *alba* in the phylogenetic tree based on the CP genome (**Figure 4**). The classification of *M. serrata* requires more samples and further investigation using molecular evidence.

Indian mulberry (*M. indica* or *M. alba* var. *indica*) is recognized as a variety of *M. alba* (Datta, 1954; Rao and Jarvis, 1986; Zeng et al., 2015; Muhonja et al., 2020). The first *Morus* CP genome (GenBank: DQ226511, 158,484 bp) was identified in *M. indica* with the reference genome method using plastid genomic DNA (Ravi et al., 2006). In this study, the CP genomes of 12 different samples, including *M. indica* var. kanva2, were *de novo* assembled and found to have identical sequences (size: 158,969 bp). Sample SL2 in group TWO, native to Sri Lanka (Xia et al., 2022), may be a hybrid progeny of *M. alba* var. *indica*. *M. indica* was clustered with *M. alba* var. *multicaulis*, indicating a close phylogenetic relationship with this taxon (**Figure 4**). Additional molecular evidence of the phylogenetic relationships of *M. indica* was provided (Muhonja et al., 2020). Therefore, *M. indica* may be derived from *M. alba* var. *multicaulis*.

In the present study, we *de novo* assembled 123 *Morus* CP genomes and found that they are highly conserved. Many *Morus* CP genomes displayed identical sequences, which indicated that they shared common maternal ancestors. We propose that the *Morus* genus includes six species, namely, *M. notabilis*, *M. celtidifolia*, *M. nigra*, *M. rubra*, *M. serrata*, and *M. alba* comprising two subspecies, *M. alba* var. *alba* and *M. alba* var. *multicaulis*. The Japanese cultivated germplasms were derived from *M. alba* var. *multicaulis*. Our findings provide valuable information for studies on the classification, domestication, and breeding improvement of mulberry.

## Data availability statement

The raw sequence data were deposited in the CNGB Sequence Archive of the China National GeneBank Database (CNGBdb) under accession number CNP 0001407.

## Author contributions

QZ, NH, and ZX conceived the project and designed the experiments. QZ assembled, annotated, and analyzed the genomes, QZ wrote the manuscript. MC, SW and XX contributed materials and isolated DNA and analyzed the data. TL helped QZ to analyze the data. All authors contributed to the article and approved the submitted version.

## Funding

This work was funded by the National Key Research and Development Program (No. 2018YFD1000602) and the Chongqing Research Program of Basic Research and Frontier Technology (cstc2021yszx-jcyj0004).

## Acknowledgments

We are grateful to Professor Elizabeth Makings from Arizona State University for kindly providing leaves of *M. celtidifolia*. We thank for the help of Ni Yang and Li Jingling from the Institute of Medicinal Plant Development for their suggestion on the annotation and analysis of chloroplast genomes. We also thank the reviewers for helpful comments on the manuscript.

## Conflict of interest

The authors declare that the research was conducted in the absence of any commercial or financial relationships that could be construed as a potential conflict of interest.

## Publisher’s note

All claims expressed in this article are solely those of the authors and do not necessarily represent those of their affiliated organizations, or those of the publisher, the editors and the reviewers. Any product that may be evaluated in this article, or claim that may be made by its manufacturer, is not guaranteed or endorsed by the publisher.
